# Association Between Albumin‐Corrected Anion Gap and Prevalent Prediabetes or Diabetes Mellitus: A Cross‐Sectional Analysis of NHANES 2005–2010

**DOI:** 10.1155/jdr/3632830

**Published:** 2026-09-18

**Authors:** Rui He, Yingjie Su, Jie Jiang, Songqing Wei, Fang Chen

**Affiliations:** ^1^ Department of Emergency Medicine, The Affiliated Changsha Central Hospital, Hengyang Medical School, University of South China, Changsha, Hunan, China, usc.edu.cn; ^2^ Department of Pediatrics, The Affiliated Changsha Central Hospital, Hengyang Medical School, University of South China, Changsha, Hunan, China, usc.edu.cn

**Keywords:** ACAG, cross-sectional study, DM, NHANES, prediabetes

## Abstract

**Objective:**

The objective of this study was to explore the cross‐sectional association between albumin‐corrected anion gap (ACAG) and the composite outcome of prediabetes or diabetes mellitus (PD‐DM) using data from the National Health and Nutrition Examination Survey (NHANES) 2005–2010.

**Methods:**

A cross‐sectional analysis was conducted, comprising 3937 adult participants aged ≥ 20 years, who were categorized into two groups: those with PD‐DM and those without PD‐DM. The baseline characteristics were compared using the most appropriate statistical tests. Logistic regression analyses, restricted cubic spline (RCS), subgroup analyses, interaction tests, and sensitivity analyses were performed to investigate the cross‐sectional relationship between ACAG and PD‐DM. Net reclassification improvement (NRI) and integrated discrimination improvement (IDI) were computed to assess whether ACAG offered incremental predictive value for PD‐DM.

**Results:**

Participants with PD‐DM exhibited higher ACAG levels (15.02 vs. 14.14, *p* < 0.01). Each unit increase in ACAG was associated with 1.16‐fold higher odds of PD‐DM after full adjustment (odds ratio [OR] = 1.16, 95% confidence interval [CI]: 1.09–1.23; *p* < 0.01). RCS showed that ACAG was linearly associated with PD‐DM (*p* for nonlinearity = 0.48). Subgroup analyses revealed a stronger association in females and lower poverty income ratio (PIR) groups (*p* for interaction < 0.05). NRI and IDI analyses confirmed that incorporating ACAG into the baseline model significantly improved risk discrimination and reclassification for PD‐DM (all *p* < 0.01). Sensitivity analyses confirmed robustness.

**Conclusion:**

This cross‐sectional study demonstrated that ACAG is positively associated with PD‐DM, especially among females and lower PIR participants. ACAG also showed significant incremental predictive value for PD‐DM risk prediction. Because of the cross‐sectional design, causal inference cannot be established. Further large‐scale prospective studies are still needed to elucidate the role of ACAG in the development of PD‐DM.

## 1. Introduction

Diabetes mellitus (DM) is a chronic metabolic disorder characterized by persistent hyperglycemia, arising from cellular insulin resistance (IR) and/or insufficient insulin secretion from pancreatic *β*‐cells [1]. Current global estimates reveal that 537 million adults are living with DM, a figure projected to exceed 783 million over the next two decades [1, 2]. Notably, the diagnosis rate among individuals under 40 has increased by 28% since 2015 [1, 3, 4]. This trend underscores the growing burden of DM, which is nearing epidemic levels. It is placing a significant strain on healthcare systems worldwide. In the United States alone, people with DM account for over 17 million emergency department visits and 8 million hospital admissions annually [5]. Persistent hyperglycemia induces progressive damage across multiple biological systems. This damage manifests as renal impairment, vision loss, peripheral neuropathy, and accelerated cardiovascular deterioration [6]. Alarmingly, more than half of global DM cases remain undiagnosed. This is particularly true in rural regions where screening accessibility lags approximately 40% behind urban centers [7]. This diagnostic gap exacerbates the risk of life‐threatening complications, highlighting the urgent need for effective and cost‐efficient strategies to improve comprehensive DM screening [7].

Current risk stratification tools primarily rely on traditional biomarkers such as glycated hemoglobin A1c (HbA1c) and fasting glucose [8, 9]. However, emerging evidence suggests that parameters of acid‐base homeostasis may provide complementary prognostic value [10, 11]. Serum anion gap (SAG), a calculated measure reflecting unmeasured anions, has been associated with IR in cohort studies [12]. Mechanistically, elevated SAG is thought to reflect increased circulating unmeasured organic anions, which may be involved in glucose metabolism independent of clinical metabolic acidosis and overt pH derangement [11]. Experimental studies support this link: An RCT confirmed that elevated lactate and raised anion gap could induce IR without altering blood pH or triggering metabolic acidosis [13]. Similarly, dietary patterns including low or high carbohydrate intake can elevate anion gap in healthy individuals in the absence of acid‐base pathology, and such subclinical anion gap perturbation is closely correlated with dysregulated glucose homeostasis [14].

Notably, conventional SAG calculations have limitations in hypoalbuminemic states, which are common in metabolic syndrome [15, 16]. The albumin‐corrected anion gap (ACAG) addresses this limitation through mathematical adjustment and demonstrates a stronger association with organic anion accumulation and tissue metabolic perturbation, especially in individuals with decreased albumin [17, 18]. ACAG serves as an integrative biomarker reflecting acid‐base homeostasis, electrolyte balance, and renal function—pathways individually linked to IR and *β*‐cell dysfunction. Importantly, ACAG is not merely a surrogate for dietary patterns (e.g., plant‐based and potassium‐rich diets); it is associated with cumulative metabolic disturbances beyond diet, including interindividual variations in acid‐base metabolism, renal acid‐handling capacity, and inflammatory status [19]. Despite the biological plausibility of this association, no large‐scale epidemiological study has specifically examined the cross‐sectional association between ACAG and the composite outcome of prediabetes or diabetes mellitus (PD‐DM) across diverse demographics. To address this gap, we conducted a cross‐sectional study using nationally representative National Health and Nutrition Examination Survey (NHANES) data the aimed at systematically investigating the relationship between ACAG and the prevalence of PD‐DM.

## 2. Materials and Methods

### 2.1. Study Design and Population

The data analyzed in this investigation were sourced from the 2005–2010 cycles of NHANES. This restricted time window was determined by multiple data availability constraints. First, the 2011–2014 cycles did not measure C‐reactive protein (CRP), an essential covariate to control for inflammatory confounding. Second, the 1999–2004 and 2017–2018 cycles lacked the WTSOG2YR sampling weight required for weighted analyses consistent with NHANES complex survey design. Third, cycles from 2019 onward were excluded to avoid potential confounding effects of the COVID‐19 pandemic on dietary behaviors and metabolic health outcomes. NHANES is a nationally representative surveillance program administered under the oversight of the United States Centers for Disease Control and Prevention. This cross‐sectional study systematically monitors population health status and dietary patterns through biennial assessments. Ethical clearance for the NHANES protocol was secured from the Institutional Review Board at the National Center for Health Statistics. All enrolled participants provided documented informed consent prior to data collection. Comprehensive details on ethical considerations and participant consent procedures are accessible via the official NHANES online portal.

**Figure 1:**
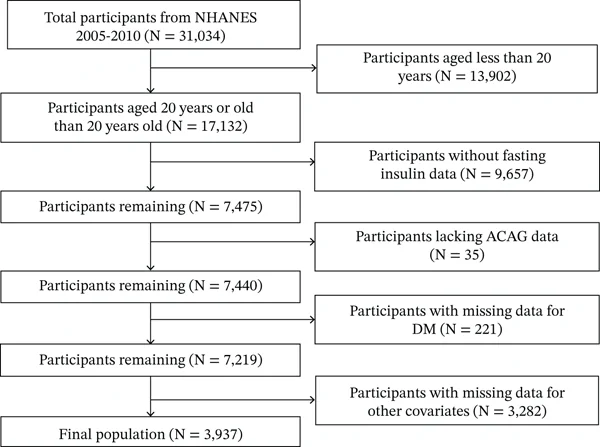
Study participant selection flowchart. Abbreviations: DM, diabetes mellitus; NHANES, National Health and Nutrition Examination Survey.

Of the 31,034 individuals who responded to the 2005–2010 NHANES surveys, 17,132 were aged ≥ 20 years and constituted the source population for this analysis. Exclusion criteria were applied sequentially. First, 9657 subjects missing fasting insulin measurements were omitted. Second, 35 subjects lacking ACAG data were deleted. Third, 221 individuals lacking DM status data were removed. Additionally, 3282 cases with incomplete covariate information were further excluded. After these exclusions, 3937 participants remained in the final analysis, representing 22.98% of the eligible adult population (≥ 20 years). The comprehensive selection workflow is visualized in Figure 1.

### 2.2. Transparency and Registration

This study involves a secondary analysis of publicly available data from the NHANES. As this is an observational cross‐sectional study utilizing deidentified public‐use data, the analysis plan was not preregistered in a public registry. However, to ensure transparency and rigor, this study was conducted and reported in accordance with the STROBE (Strengthening the Reporting of Observational Studies in Epidemiology) guidelines for cross‐sectional studies. The analytical plan was defined a priori based on the existing literature, and all methods were executed as described.

### 2.3. Ascertainment of PD‐DM

The diagnostic criteria for DM included any of the following indicators: prescribed hypoglycemic agents or insulin therapy, physician‐confirmed clinical diagnosis, fasting plasma glucose ≥ 7.0 mmol/L, HbA1c level ≥ 6.5%, or postprandial glucose ≥ 11.1 mmol/L following standardized oral glucose challenge [20]. Prediabetes was defined as the presence of impaired fasting glucose (fasting plasma glucose 6.1–6.9 mmol/L) and/or impaired glucose tolerance (2‐h plasma glucose 7.8–11.0 mmol/L following a 75‐g oral glucose tolerance test) [21]. PD‐DM included participants diagnosed with either PD‐DM.

### 2.4. Measurement of ACAG

The anion gap (AG, mmol/L) was derived using the formula AG = (Na^+^ + K^+^) − (Cl^−^ + HCO3^−^). To account for albumin variability, ACAG (mmol/L) was computed as ACAG = [4.4 − serum albumin concentration (g/dL)] × 2.5 + AG [22]. These calculations align with established methodologies for evaluating acid‐base and electrolyte imbalances in clinical biochemistry.

### 2.5. Covariates

The study gathered participants′ demographic details, behavioral risk factors, laboratory parameters, and comorbidities. Demographics covered age, sex, ethnicity, education, body mass index (BMI), and poverty income ratio (PIR). Behavioral factors included smoking, alcohol use, calorie, potassium, and protein intake, and physical activity (PA) levels. Laboratory parameters comprised CRP, fasting insulin, albumin, and serum electrolytes (Na^+^, K^+^, Cl^−^, HCO3^−^). To reduce multicollinearity in multivariate models, albumin and electrolyte levels were used solely for ACAG calculations and then excluded from later analyses. Comorbidity assessment identified pre‐existing conditions including chronic kidney disease (CKD), hyperlipidemia, and hypertension. The definition of covariates is provided in Table S1.

### 2.6. Statistical Analysis

Statistical analyses were conducted using R Version 4.4.3 (R Foundation for Statistical Computing). To align with the multistage survey methodology of the NHANES, weighted analyses incorporating the WTSOG2YR sampling weights were applied to characterize baseline participant demographics. Numeric variables are reported as weighted mean values with standard error. Categorical data are presented as adjusted proportions (%) to reflect population representativeness. Between‐group comparisons for continuous and categorical data were evaluated using Student′s *t*‐tests and Pearson′s chi‐square tests, respectively.

Logistic regression models (both unadjusted and multivariable‐adjusted) were employed to assess relationships between ACAG and PD‐DM outcomes. Variance inflation factor (VIF) was used to assess multicollinearity; all covariates had VIF < 2, indicating no significant multicollinearity. In multivariable analyses, ACAG was examined as either a continuous parameter or quartile‐stratified categorical variable across sequential adjustment tiers: Crude model, unadjusted; Model 1, adjusted for demographic factors (age, sex, race, PIR, and educational attainment); Model 2, further adjusted for anthropometric and lifestyle factors (BMI, energy intake [log‐transformed, kcal/day], potassium intake [mg/day], protein intake [g/day], alcohol use, smoking status, PA [log‐transformed]); Model 3, additionally adjusted for clinical biomarkers and comorbidities (CRP, hypertension, hyperlipidemia, and CKD). Effect estimates were expressed as adjusted odds ratio (OR) with 95% confidence interval (CI). Statistical significance was defined as two‐tailed *p* values < 0.05.

We further calculated continuous and categorical net reclassification improvement (NRI) and integrated discrimination improvement (IDI) to quantify the incremental predictive value of ACAG for PD‐DM risk prediction.

To investigate heterogeneity in the associations between ACAG and PD‐DM, stratified analyses were performed across clinically relevant subgroups. These subgroups were defined by the following variables: age (20–45 years, 46–60 years, and > 60 years); biological sex; race (White, Black, Mexican‐American, and other); PIR (low, middle, and high); educational attainment (below high school, high school/equivalent, and postsecondary); smoking status (never, former, and current); PA quartiles; hypertension status; hyperlipidemia status; and CKD status.

To evaluate the variability of associations across different subgroups, tests for interaction were incorporated into the analysis. Sensitivity analyses further validated the robustness of primary findings. A two‐tailed *p* value threshold of < 0.05 defined statistical significance.

## 3. Results

### 3.1. Participant Characteristics

Of the 3937 participants included in the study, 2122 (51.35%) were male and 1815 (48.65%) were female, with a mean age of 45.02 ± 0.48 years. The prevalence of PD‐DM was 36.53% among all participants. Participants with PD‐DM were more likely to have certain distinctive characteristics relative to those without PD‐DM. They were more likely to be of Mexican and other races, have lower PIR, lower educational attainment, lower energy intake, lower alcohol consumption, lower albumin, lower chloride, lower potassium, and lower PA levels. They also had higher BMI, higher fasting insulin levels, higher CRP levels, and a higher prevalence of chronic medical conditions (CKD, hyperlipidemia, and hypertension). Additionally, they had higher ACAG (all *p* < 0.05). Although albumin levels differed significantly across groups and were used to calculate ACAG, albumin itself was not included as an independent covariate in the multivariable regression models to avoid collinearity with ACAG. The clinical and biochemical characteristics of participants with and without PD‐DM are presented in Table 1. The comparison of baseline characteristics between excluded and included populations is detailed in Table S2.

**Table 1:** Baseline characteristics of participants in NHANES 2005–2010.

Characteristics	Total *n* = 3937	Nonprediabetes and DM *n* = 2499	Prediabetes and DM *n* = 1438	*p*
Age, year	45.02 (0.48)	41.37 (0.51)	53.74 (0.65)	< 0.01
Gender, *n* (%)				0.01
Male	2122 (51.35)	1281 (68.26)	841 (31.74)	
Female	1815 (48.65)	1218 (72.81)	597 (27.19)	
Race, *n* (%)				0.76
White	2102 (73.96)	1341 (70.62)	761 (29.38)	
Black	695 (9.86)	452 (71.20)	243 (28.80)	
Mexican	658 (6.79)	391 (67.89)	267 (32.11)	
Other	482 (9.39)	315 (70.42)	167 (29.58)	
BMI, kg/m^2^	28.28 (0.13)	27.21 (0.15)	30.83 (0.20)	< 0.01
Education				< 0.01
Less than high school	862 (14.26)	462 (61.63)	400 (38.37)	
High school or equivalent	940 (23.08)	570 (66.39)	370 (33.61)	
Greater than high school	2135 (62.66)	1467 (73.99)	668 (26.01)	
PIR^a^, *n* (%)				< 0.01
Low	1014 (16.06)	614 (68.26)	400 (31.74)	
Middle	1476 (34.80)	898 (67.41)	578 (32.59)	
High	1447 (49.14)	987 (73.37)	460 (26.63)	
Energy, kcal/day	8.24 (0.01)	8.26 (0.01)	8.20 (0.02)	< 0.01
Potassium, mg/day	5278.43 (52.34)	5286.78 (62.59)	5258.49 (83.81)	0.78
Protein, mg/day	163.80 (1.49)	165.13 (1.64)	160.64 (2.80)	0.16
PA, met	7.08 (0.05)	7.13 (0.07)	6.96 (0.05)	0.03
Drinking, drink/day				< 0.01
Never	402 (8.77)	209 (57.41)	193 (42.59)	
Former	708 (14.45)	372 (62.34)	336 (37.66)	
Mild	1373 (37.07)	891 (70.82)	482 (29.18)	
Moderate	587 (16.64)	426 (76.20)	161 (23.80)	
Heavy	867 (23.07)	601 (75.86)	266 (24.14)	
Smoking				< 0.01
Former	1044 (25.90)	565 (61.42)	479 (38.58)	
Never	2069 (52.42)	1365 (72.52)	704 (27.48)	
Current	824 (21.68)	569 (76.33)	255 (23.67)	
ACAG, mmol/L	14.40 (0.08)	14.14 (0.09)	15.02 (0.08)	< 0.01
Albumin, g/dL	4.26 (0.01)	4.28 (0.01)	4.22 (0.01)	< 0.01
Potassium, mmol/L	4.02 (0.01)	4.01 (0.01)	4.06 (0.01)	0.01
Chloride, mmol/L	104.10 (0.10)	104.36 (0.11)	103.49 (0.12)	< 0.01
Bicarbonate, mmol/L	25.02 (0.09)	25.02 (0.09)	25.00 (0.12)	0.79
Sodium, mmol/L	139.14 (0.09)	139.21 (0.10)	139.00 (0.11)	0.06
Insulin, uU/mL	11.45 (0.23)	9.74 (0.24)	15.54 (0.41)	< 0.01
CRP, mg/dL	0.37 (0.01)	0.30 (0.02)	0.52 (0.02)	< 0.01
Hypertension				< 0.01
No	2481 (68.59)	1878 (80.73)	603 (19.27)	
Yes	1456 (31.41)	621 (48.08)	835 (51.92)	
CKD				< 0.01
No	3384 (89.84)	2306 (73.85)	1078 (26.15)	
Yes	553 (10.16)	193 (40.60)	360 (59.40)	
Hyperlipidemia				< 0.01
No	1042 (28.54)	829 (84.82)	213 (15.18)	
Yes	2895 (71.46)	1670 (64.75)	1225 (35.25)	
Year cycles				0.24
2005–2006	1164 (34.31)	754 (71.52)	410 (28.48)	
2007–2008	1324 (32.99)	775 (67.55)	549 (32.45)	
2009–2010	1449 (32.70)	970 (72.33)	479 (27.67)	

*Note:* Continuous variables are expressed as unweighted mean (weighted standard deviation); categorical variables are expressed as unweighted count (weighted percentage).

Abbreviations: ACAG, albumin‐corrected anion gap; BMI, body mass index; CKD, chronic kidney disease; CRP, C‐reactive protein; DM, diabetes mellitus; PA, physical activity; PIR, poverty income ratio.

^a^PIR was categorized into three groups: low‐income (< 1.3), middle‐income (1.3–3.5), and high‐income (> 3.5).

### 3.2. Association of ACAG With PD‐DM

In this study, ACAG was examined through both continuous‐scale assessment and categorical stratification using quartile‐based groupings. Multivariable logistic regression analyses revealed a consistent positive association between ACAG levels and the odds of PD‐DM (Table 2). In the crude model, each unit increase in ACAG was associated with 1.30‐fold higher odds of PD‐DM (OR = 1.30, 95% CI: 1.23–1.36, *p* < 0.01). After adjusting for age, gender, race, socioeconomic status (PIR), and education (Model 1), the association remained robust (OR = 1.25, 95% CI: 1.19–1.31, *p* < 0.01). Further adjustment for BMI, dietary factors (energy, protein, and alcohol intake), smoking, and PA (Model 2) slightly attenuated the effect (OR = 1.19, 95% CI: 1.13–1.26, *p* < 0.01). In the fully adjusted model (Model 3), which incorporated inflammatory markers (CRP), hypertension, hyperlipidemia, and CKD, the association persisted (OR = 1.16, 95% CI: 1.09–1.23, *p* < 0.01).

**Table 2:** Association between ACAG and PD‐DM in different models.

	OR (95% CI), *p*			
	Crude model	Model 1	Model 2	Model 3
ACAG (continuous)	1.30 (1.23, 1.36) < 0.01	1.25 (1.19, 1.31) < 0.01	1.19 (1.13, 1.26) < 0.01	1.16 (1.09, 1.23) < 0.01
ACAG (quartiles)				
Q1	Reference	Reference	Reference	Reference
Q2	1.80 (1.41, 2.30) < 0.01	1.77 (1.32, 2.37) < 0.01	1.71 (1.26, 2.32) < 0.01	1.62 (1.18, 2.24) < 0.01
Q3	1.88 (1.48, 2.40) < 0.01	1.76 (1.34, 2.31) < 0.01	1.51 (1.13, 2.02) 0.01	1.42 (1.05, 1.90) 0.02
Q4	3.29 (2.58, 4.18) < 0.01	2.72 (2.13, 3.46) < 0.01	2.26 (1.76, 2.92) < 0.01	1.99 (1.51, 2.63) < 0.01
*p* for trend	< 0.01	< 0.01	< 0.01	< 0.01

*Note:* ACAG quartile cutoff values: Q1 ≥ 13.45, 13.45 < Q2 ≤ 14.65, 14.65 < Q3 ≤ 15.90, Q4 > 15.90. Crude model: unadjusted. Model 1: adjusted for demographic factors: age, gender, race, PIR, and education level. Model 2: adjusted for all variables in Model 1 plus anthropometric and lifestyle factors: BMI, energy intake (log‐transformed, kcal/day), potassium intake (mg/day), protein intake (g/day), alcohol use, smoking status, and PA (log‐transformed). Model 3: adjusted for all variables in Model 2 plus clinical biomarkers and comorbidities: CRP, hypertension, hyperlipidemia, and CKD.

Abbreviations: 95% CI, 95% confidence interval; ACAG, albumin‐corrected anion gap; BMI, body mass index; CKD, chronic kidney disease; CRP, C‐reactive protein; DM, diabetes mellitus; OR, odds ratio; PA, physical activity; PIR, poverty income ratio.

When stratified by ACAG quartiles, a dose‐dependent relationship was observed. Compared with the lowest quartile (Q1), participants in the highest quartile (Q4) exhibited 3.29‐fold higher odds in the crude model (OR = 3.29, 95% CI: 2.58–4.18, *p* < 0.01). This gradient remained significant even after full adjustment (Model 3: OR = 1.99, 95% CI: 1.51–2.63, *p* < 0.01). A linear trend was confirmed across all models (*p* for trend < 0.01). Q2, Q3, and Q4 showed significantly higher odds than Q1 across all models. Although Q3 had a marginally higher OR than Q2 in the crude model (1.88 vs. 1.80), this pattern reversed after adjustment, with Q3 estimates falling below Q2 in Models 1–3. The stepwise attenuation of ORs with additional adjustment suggests partial mediation by metabolic and inflammatory factors. These findings suggest that ACAG is independently associated with PD‐DM, with higher ACAG levels showing increased odds of the conditions.

We further evaluated whether adding ACAG to the fully adjusted baseline model improved risk prediction for PD‐DM by calculating categorical NRI, continuous NRI, and IDI (Table S3). Relative to the baseline model without ACAG, inclusion of ACAG significantly enhanced risk stratification and discrimination. The categorical NRI was 0.08 (95% CI: 0.06–0.11, *p* < 0.01), continuous NRI was 0.42 (95% CI: 0.35–0.48, *p* < 0.01), and IDI was 0.04 (95% CI: 0.03–0.06, *p* < 0.01).

**Figure 2:**
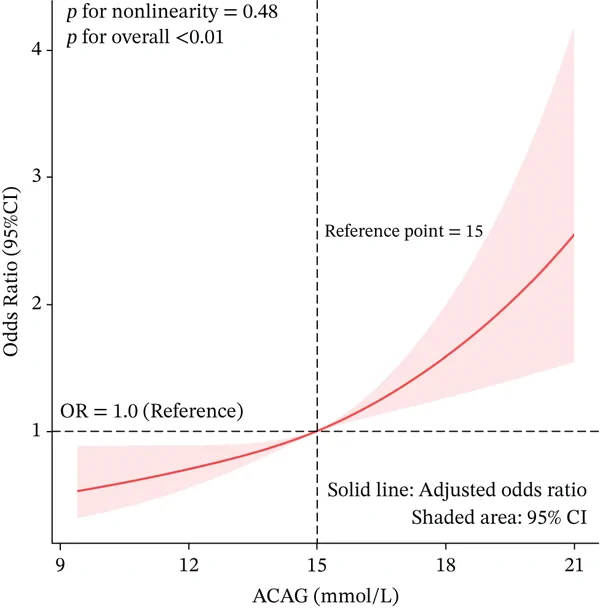
RCS analysis showing the dose–response relationship between ACAG and PD‐DM. The solid line represents the multivariable‐adjusted OR, and the shaded area indicates the 95% CI. The horizontal dashed line denotes OR = 1.0 (null effect), and the vertical dashed line indicates the reference point (15 mmol/L). The *p* value for nonlinearity was 0.48, and the overall association was statistically significant (*p* < 0.01). Abbreviations: ACAG, albumin‐corrected anion gap; DM, diabetes mellitus; OR, odds ratio; 95% CI, 95% confidence interval.

The restricted cubic spline (RCS) modeling, with three knots placed at the 10th, 50th, and 90th percentiles of the ACAG distribution and 15 mmol/L set as the reference value revealed, after covariate adjustment, a significant linear association. No statistically significant nonlinear relationship was observed (*p* for nonlinearity = 0.48). Higher ACAG was associated with increased odds of PD‐DM in a dose–response pattern (Figure 2).

### 3.3. Subgroup Analyses and Sensitivity Analyses

**Figure 3:**
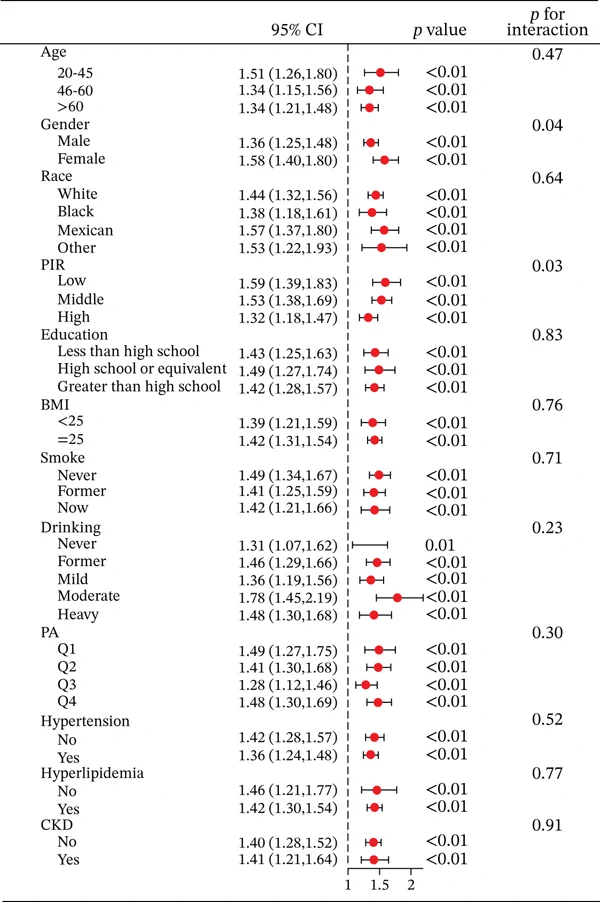
Subgroup analysis of the association between ACAG and PD‐DM. Multivariable‐adjusted OR and 95% CI are presented. *p* values for interaction test the heterogeneity of associations across subgroups. Abbreviations: ACAG, albumin‐corrected anion gap; DM, diabetes mellitus; PIR, poverty income ratio; BMI, body mass index; PA, physical activity; CKD, chronic kidney disease; OR, odds ratio; 95% CI, 95% confidence interval.

Subgroup analyses were conducted to evaluate potential modifying effects on the relationship between ACAG and PD‐DM. A consistent and robust association between elevated ACAG levels and PD‐DM was identified across all subgroups categorized by age, gender, race, PIR, education, drinking status, smoking status, PA, CKD, hyperlipidemia, and hypertension (all *p* < 0.05). Effect modification analyses showed no statistically significant subgroup differences for the association between ACAG and PD‐DM across strata of age, race, education level, BMI, drinking status, smoking status, PA levels, CKD, hyperlipidemia, and hypertension (all interaction *p* > 0.05). These findings suggest that the positive relationship between ACAG and PD‐DM remains stable regardless of variations in these demographic, socioeconomic, or clinical factors. However, significant effect modifications were observed for gender and PIR (interaction *p* values < 0.05) (Figure 3).

To investigate gender‐specific and socioeconomic (PIR) influences on the association between ACAG and PD‐DM, subgroup analyses incorporating fitted curves were conducted. Results indicated a heightened association strength in females relative to males (Figure S1). Similarly, comparisons across PIR strata demonstrated significantly stronger associations in low and middle income groups compared with high income populations (Figure S2). These findings suggest that both biological (gender) and socioeconomic (PIR) factors may modulate the ACAG‐glycemic dysregulation linkage.

Furthermore, given the stronger association observed in females, we further performed stratified analyses restricted to female participants to explore whether menopausal status and age range modified the ACAG and PD‐DM relationship (Table S4). Although statistical significance was reached only in menopausal women and those aged ≥ 45 years (consistent with greater statistical power in these larger subgroups), the effect estimates were directionally similar across all strata, and the *p* values for interaction were far from significant (0.50 and 0.90), indicating no effect modification by menopausal status or age.

To support our conclusions, we conducted sensitivity analyses. After excluding participants with prediabetes (*n* = 781), the association between ACAG and DM remained robust across all models (Table S5). Specifically, in the fully adjusted model (Model 3), each one‐unit increase in ACAG was associated with 26% higher odds of DM (OR = 1.26, 95% CI: 1.17–1.36, *p* < 0.01), with a significant dose–response relationship across quartiles (*p* for trend < 0.01). Similarly, after excluding participants with DM (*n* = 657), the association between ACAG and prediabetes remained significant but with a smaller effect size (Table S6). In Model 3, each one‐unit increase in ACAG was associated with 11% higher odds of prediabetes (OR = 1.11, 95% CI: 1.03–1.20, *p* = 0.01). The dose–response relationship was also evident (*p* for trend = 0.01); however, the highest quartile (Q4) showed a weaker association (OR = 1.74, 95% CI: 1.23–2.45) compared with that observed for DM (OR = 2.55, 95% CI: 1.84–3.54). RCS consistently revealed strong associations between elevated ACAG levels and both DM and prediabetes (all *p* < 0.05) (Figure S3 and S4).

## 4. Discussion

In this cross‐sectional study of 3937 US adults, we observed a significant positive association between ACAG and the prevalence of PD‐DM. Crucially, there was a linear positive relationship between ACAG levels and the odds of PD‐DM, even after comprehensive adjustment for confounders. Participants in the highest ACAG quartile had 1.99‐fold higher odds of PD‐DM compared with those in the lowest quartile (95% CI: 1.51–2.63). NRI and IDI analyses revealed ACAG added significant predictive value for PD‐DM risk beyond conventional covariates. Subgroup and sensitivity analyses confirmed the robustness of these findings. Importantly, interaction assessments indicated that gender and PIR might be key factors modifying the association between ACAG and PD‐DM.

The observed association aligns with biological mechanisms linking organic anion accumulation to glucose dysregulation. Experimental evidence demonstrates that isolated elevation of organic anions and anion gap, independent of systemic pH changes or metabolic acidosis, may disrupt insulin signaling [23] which is potentially mediated by reduced insulin receptor binding [24], possible inhibition of downstream signaling pathways [25, 26], and subsequent reduction in insulin sensitivity [13]. Concurrently, acidosis‐induced cortisol secretion may aggravate IR in skeletal muscle and adipose tissue [27, 28], whereas low pH environments potentially downregulate insulin‐sensitizing hormones [11, 29] and may suppress leptin secretion [30]. Relevant human studies further suggest biological plausibility that organic anion overload may be linked to impaired glucose metabolism: An RCT showed that elevated lactate and higher anion gap were associated with IR in healthy participants without inducing pH disturbance or metabolic acidosis, suggesting that organic anion overload itself may impair glucose metabolism [13]. Another cross‐sectional study found that anion gap elevation can occur in healthy individuals under different dietary patterns, whereas elevated anion gap in metabolically abnormal populations is independently correlated with dysregulated glucose homeostasis (elevated HOMA‐IR, HOMA‐*β*, and C‐peptide levels) [31]. Furthermore, organic anion elevation (measured by plasma lactate) was found to be associated with IR independent of obesity, and short‐term overfeeding (which increases dietary acid load) could reduce insulin sensitivity and elevate plasma lactate [19]. As ACAG is mathematically derived from serum bicarbonate and albumin—both of which reflect systemic acid‐base status—elevated ACAG may serve as a clinical indicator of metabolic perturbation and organic anion overload that is associated with these mechanistic pathways. Collectively, these interconnected pathways may contribute to glucose metabolism dysregulation and are associated with IR and DM.

Notably, the association between ACAG dysregulation and glycemic disorder may be bidirectional. On the one hand, disturbances in acid‐base balance are linked to IR and hyperglycemia. On the other hand, hyperglycemia could disrupt systemic acid‐base homeostasis and thereby increase ACAG concentrations. Hyperglycemia‐triggered osmotic diuresis promotes urinary electrolyte wasting and lowers serum bicarbonate levels, driving metabolic acidosis and a widened anion gap [32, 33]. In diabetic states, buildup of organic anions such as lactate, beta‐hydroxybutyrate, and acetoacetate contributes to an increased anion gap, and the rise in anion gap typically corresponds to the decline in plasma bicarbonate [33]. These reciprocal mechanisms indicate that higher ACAG could be both a risk marker of impaired glucose metabolism and a metabolic consequence driven by disrupted glycemic homeostasis.

Animal models reveal that acidic shifts in interstitial fluid pH within ascitic and metabolic tissues may precede clinical DM manifestations [34]. Acidic shifts in interstitial pH may impair insulin–receptor binding affinity and hinder cellular glucose absorption [27]. These mechanistic findings are consistent with human evidence [12]. Data from the NHANES 1999–2002 survey showed that among healthy adults, those with serum bicarbonate concentrations below 22 mmol/L were associated with fasting insulin values approximately 12.7 pmol/L higher than counterparts with bicarbonate exceeding 25 mmol/L [35]. Interventional studies further suggest that alkali therapy may improve IR in acidotic patients [36], whereas acute acid loading in healthy volunteers may reduce glucose disposal and cellular insulin action [37]. Additionally, low urinary pH—a noninvasive indicator of systemic acid retention—has been consistently associated with higher IR in community‐based cohorts [38, 39]. These human studies suggest that even modest acid‐base disturbances may be clinically relevant to glycemic dysregulation.

We acknowledge that ACAG may partially reflect a healthy, plant‐based, potassium‐rich dietary pattern, which is associated with lower DM risk [40]. Plant‐based diets are typically alkalizing and rich in potassium, both of which can influence acid‐base balance and, consequently, the anion gap [41, 42]. However, several lines of evidence suggest ACAG is not simply a dietary surrogate. First, we adjusted for dietary energy, potassium intake, and protein intake in our multivariable models, yet the association between ACAG and PD‐DM remained significant. Second, a randomized controlled trial found that a 6‐week healthy diet did not improve insulin sensitivity compared with a Western diet [43], suggesting that metabolic outcomes are not solely determined by dietary pattern. Third, low carbohydrate intake was associated with elevated anion gap and IR in lean individuals [31], indicating that ACAG can rise even in contexts not typically classified as “unhealthy” diets. From a clinical perspective, ACAG offers practical advantages over detailed dietary assessments: It is inexpensive, routinely available from standard electrolyte panels, and free from recall bias, making it potentially valuable for DM risk stratification in resource‐limited settings.

Our analysis of 3937 adults revealed a significant positive linear association between ACAG levels and PD‐DM, with a PD‐DM prevalence of 36.53% in our study population. Participants in the highest quartile had 3.29‐fold higher odds compared with those in the lowest quartile (95% CI: 2.58–4.18), which remained elevated at 1.99‐fold (95% CI: 1.51–2.63) in fully adjusted models. Consistent with a prior nested case–control study of 630 women, higher plasma bicarbonate levels were associated with 24% lower DM incidence (OR = 0.76), with a significant dose–response trend (*p* = 0.04) [44]. Similarly, a randomized trial in 145 DM patients with CKD reported that serum bicarbonate was associated with IR, and bicarbonate supplementation was associated with improvements in IR [36]. In a cross‐sectional study of healthy individuals, higher dietary acid load was associated with acid‐base dysregulation and IR [19]. This is consistent with our findings that ACAG, as a marker of organic anion metabolic disturbance, is associated with PD‐DM and is further supported by studies showing that dietary acid load‐induced metabolic acidosis is closely linked to IR [19]. However, evidence is not entirely uniform; another cross‐sectional study in CKD patients found that although dietary acid load was correlated with serum bicarbonate, no significant association with IR was observed [45], suggesting that the relationship may vary by population or be modified by underlying kidney function. Our study complements and expands existing literature by observing, for the first time in a nationally representative sample, a linear positive association between ACAG and PD‐DM.

Our study indicates that gender and PIR significantly modify the association between ACAG and PD‐DM, with females and individuals from lower PIR showing stronger associations. To further explore the biological basis of the observed sex difference and address potential confounding from menopausal transition, we examined whether menopausal status or age group modified the association among women. Neither menopausal status nor age group significantly modified the association between ACAG and PD‐DM (interaction *p* = 0.50 and 0.90, respectively). Although the OR was numerically higher in nonmenopausal women and younger women aged < 45 years, these subgroups failed to reach statistical significance, which may be attributable to the limited sample size of nonmenopausal participants. The consistent direction of effect across strata suggests that the stronger ACAG‐related glycemic risk among females is unlikely to be fully explained by perimenopausal estrogen fluctuations or age‐related metabolic transitions alone. These findings provide preliminary hypotheses for potential alternative mechanisms underlying the sex difference. Prior evidence suggests that sex chromosome polymorphisms and baseline estrogen signaling—rather than transient transitional hormonal shifts during the menopausal transition—may disrupt carbohydrate and lipid homeostasis, potentially increasing susceptibility to DM [46]. Estrogen receptors are expressed in pancreatic *β*‐cells, and estrogen signaling exerts protective effects against glucotoxic *β*‐cell injury [47]. Moreover, a prospective, nested case–control study has reported a link between lower plasma bicarbonate levels and a higher risk of DM in women [44]; consistently, our stratified analyses suggest that this female vulnerability may persist independent of menopausal stage. However, we acknowledge that our dichotomous classification of menopausal status and age does not permit direct identification of the perimenopausal transition, which is known to influence both acid‐base homeostasis and insulin sensitivity. Therefore, we cannot exclude the possibility that perimenopausal physiological changes contribute to the observed sex difference. The NHANES dataset lacks standardized criteria to identify perimenopausal individuals and serial reproductive hormone measurements (e.g., estradiol and FSH), precluding definitive conclusions regarding dynamic estrogen changes across the menopausal transition. Future studies incorporating detailed hormonal profiling and precise menopausal staging are needed to further clarify these mechanisms. Regarding socioeconomic disparities, a systematic review and meta‐analysis have revealed an inverse relationship between socioeconomic status and HbA1c levels in individuals with DM [48]. Consistently, research shows that lower socioeconomic status is associated with a higher prevalence of both diagnosed and undiagnosed DM, as well as a higher prevalence of DM complications [49]. This may stem from limited access to nutritious diets, reliance on high‐calorie but nutrient‐poor foods, overconsumption of energy‐dense processed foods, sedentary lifestyles, inadequate health awareness, and insufficient medical resources [50, 51]. Given these insights, future research should investigate how healthcare system factors contribute to these disparities, while also addressing gender‐specific and socioeconomic differences in metabolic dysregulation.

This study used nationally representative data to explore the link between ACAG and PD‐DM. We employed weighted adjustments for better generalizability and controlling for confounders to boost validity. However, there are limitations: First, the cross‐sectional nature of this study prevents any determination of the temporal sequence between ACAG and PD‐DM, thus precluding causal inferences. Although our hypothesis posits that elevated ACAG may contribute to glucose dysregulation, the possibility of reverse causality cannot be ruled out. For instance, DM‐related metabolic disturbances, such as ketoacidosis or early subclinical renal tubular acidosis, could directly elevate the anion gap. This inherent bidirectional ambiguity is a key limitation of our design and must be considered when interpreting the findings. Second, although multiple confounders were adjusted for, residual confounding may still exist, such as dietary acid load, medication use, and more detailed renal function markers. Third, the final analytical sample accounted for 22.98% of eligible adults aged ≥ 20 years. Compared with excluded participants, included participants were older, more likely to be male and non‐Hispanic White, and had higher BMI, educational attainment, and PIR (Table S2; all *p* < 0.01). PD‐DM prevalence was comparable between included and excluded groups (29.52% vs. 28.62%, *p* = 0.52). This finding partly reduces concerns over selection bias. Nevertheless, excluded participants contained a larger share of females (56.61% vs. 48.65%) and individuals with low PIR (27.85% vs. 16.06%). Our subgroup analyses detected stronger ACAG and PD‐DM associations in these two subgroups (interaction *p* < 0.05). For this reason, the overall effect estimates from the present study may be conservative. In addition, White participants made up 73.96% of our sample. This composition limits the generalizability of our findings to racial minorities and populations outside the United States. Fourth, our analysis was based on NHANES data from 2005–2010, which may limit the generalizability of our findings to contemporary populations. Although this dataset offered comprehensive data for our analysis, dietary patterns, obesity prevalence, and metabolic profiles may have shifted in the intervening years. Therefore, replication in more recent NHANES cycles or other contemporary cohorts would be valuable. Fifth, we recognize that without replication in independent cohorts, our findings require cautious interpretation. As this represents the first large‐scale examination of ACAG and PD‐DM using nationally representative data, we emphasize that our study establishes an epidemiological foundation for hypothesis generation rather than definitive causal inference. Future research should prioritize validation in diverse populations. Sixth, multiple subgroup interaction tests may inflate the Type I error rate. Consistent with standard epidemiological practices, unadjusted interaction *p* values were reported to avoid elevated Type II error and preserve true heterogeneity. Accordingly, subgroup findings should be interpreted cautiously. Seventh, the lack of preregistration, although mitigated by a clearly defined a priori analytical plan, may introduce potential bias, and thus our findings should be considered exploratory. In light of these limitations, future prospective longitudinal studies with diverse multinational cohorts are needed to establish the temporal sequence, clarify the directionality of the association, and determine whether ACAG serves as a risk marker or merely reflects diabetic metabolic disturbances.

## 5. Conclusion

In this cross‐sectional analysis, higher ACAG levels were independently associated with greater odds of prevalent PD‐DM, with stronger associations observed among females and individuals with lower PIR. These correlational findings warrant confirmation in prospective cohort studies to clarify potential causal pathways and temporal sequences.

**Nomenclature**
ACAG: albumin‐corrected anion gap
BMI: body mass index
CI: confidence interval
CKD: chronic kidney disease
CRP: C‐reactive protein
DM: diabetes mellitus
HbA1c: glycated hemoglobin A1c
IDI: integrated discrimination improvement
IR: insulin resistance
NHANES: National Health and Nutrition Examination Survey
NRI: net reclassification improvement
OR: odds ratio
PA: physical activity
PD‐DM: prediabetes or diabetes mellitus
PIR: poverty income ratio
RCS: restricted cubic spline
SAG: serum anion gap

## Author Contributions

Conception and design: Fang Chen, Rui He, Yingjie Su, and Jie Jiang. Administrative support: Jie Jiang, Songqing Wei, and Rui He. Provision of study materials or patients: Jie Jiang, Songqing Wei, and Rui He. Collection and assembly of data: Fang Chen, Yingjie Su, and Songqing Wei. Data analysis and interpretation: Fang Chen, Yingjie Su, and Jie Jiang. Final approval of manuscript: All authors. Rui He, Yingjie Su, Jie Jiang, and Songqing Wei have contributed to the work equally and should be regarded as cofirst authors.

## Funding

This study was funded by the Changsha Natural Science Foundation (kq2403163).

## Ethics Statement

The NHANES study protocol was approved by the United States National Center for Health Statistics Research Ethics Review Board, and written informed consent was provided by all participants. The experimental protocols involving human subjects adhered to the guidelines of national, international, and institutional regulations or the Declaration of Helsinki as stated in the manuscript.

## Consent

All participants in this study consented to publication.

## Conflicts of Interest

The authors declare no conflicts of interest.

## Supporting Information

Additional supporting information can be found online in the Supporting Information section.

## Supporting information


**Supporting Information 1** Figure S1: Gender‐stratified RCS analysis of the association between ACAG and PD‐DM. The solid lines represent multivariable‐adjusted OR for males and females separately, and the shaded areas indicate the 95% CI. The horizontal dashed line denotes OR = 1.0 (null effect), and the vertical dashed line indicates the reference point (15 mmol/L). *p* for nonlinearity = 0.48; *p* for overall = 0.02. Abbreviations: 95% CI, 95% confidence interval; ACAG, albumin‐corrected anion gap; DM, diabetes mellitus; OR, odds ratio.

**Supporting Information 2** Figure S2: PIR‐stratified RCS analysis of the association between ACAG and PD‐DM. The solid lines represent multivariable‐adjusted OR for low, middle, and high PIR groups, and the shaded areas indicate the 95% CI. The horizontal dashed line denotes OR = 1.0 (null effect), and the vertical dashed line indicates the reference point (15 mmol/L). *p* for nonlinearity = 0.48; *p* for overall < 0.01. Abbreviations: 95% CI, 95% confidence interval; ACAG, albumin‐corrected anion gap; DM, diabetes mellitus; PIR, poverty income ratio; OR, odds ratio.

**Supporting Information 3** Figure S3: RCS analysis of the association between ACAG and DM after excluding individuals with prediabetes. The solid line represents the multivariable‐adjusted OR, and the shaded area indicates the 95% CI. The horizontal dashed line denotes OR = 1.0 (null effect), and the vertical dashed line indicates the reference point (15 mmol/L). *p* for nonlinearity = 0.10; *p* for overall < 0.01. Abbreviations: 95% CI, 95% confidence interval; ACAG, albumin‐corrected anion gap; DM, diabetes mellitus; OR, odds ratio.

**Supporting Information 4** Figure S4: RCS analysis of the association between ACAG and prediabetes after excluding individuals with DM. The solid line represents the multivariable‐adjusted OR, and the shaded area indicates the 95% CI. The horizontal dashed line denotes OR = 1.0 (null effect), and the vertical dashed line indicates the reference point (15 mmol/L). *p* for nonlinearity = 0.10; *p* for overall < 0.01. Abbreviations: 95% CI, 95% confidence interval; ACAG, albumin‐corrected anion gap; OR, odds ratio.

**Supporting Information 5** Table S1: The definition of covariates.

**Supporting Information 6** Table S2: Comparison of baseline characteristics between included and excluded participants (restricted to those with available sampling weights and complete data), NHANES 2005–2010.

**Supporting Information 7** Table S3: Reclassification and discrimination indices for PD‐DM after adding ACAG to the fully adjusted baseline model.

**Supporting Information 8** Table S4: Subgroup analysis of the association between ACAG and PD‐DM according to menopausal status and age range in females.

**Supporting Information 9** Table S5: The association between ACAG and DM (*n* = 657) in different models (after excluding individuals with prediabetes [*n* = 781]).

**Supporting Information 10** Table S6: The association between ACAG and prediabetes (*n* = 781) in different models (after excluding individuals with DM [*n* = 657]).

## Data Availability

The data that support the findings of this study are available from the corresponding author upon reasonable request.
