# Supplementary material for: Association Between Albumin‐Corrected Anion Gap and Prevalent Prediabetes or Diabetes Mellitus: A Cross‐Sectional Analysis of NHANES 2005–2010
Source: J Diabetes Res. 2026 Sep 18;2026:3632830. doi: 10.1155/jdr/3632830 (PMC13589189; doi:10.1155/jdr/3632830)
Supplement: Supplementary file 1 [file JDR-2026-3632830-s004.docx]

**Table S6**. The association between ACAG and prediabetes (n=781) in different models (after excluding individuals with DM [n=657]).

|  | OR(95%CI), P-Value | | | |
| --- | --- | --- | --- | --- |
|  | Crude model | Model 1 | Model 2 | Model 3 |
| ACAG(continuous) | 1.21(1.14,1.29) <0.01 | 1.18(1.10,1.25) <0.01 | 1.14(1.06,1.22) <0.01 | 1.11(1.03,1.20) 0.01 |
| ACAG (quartiles) |  |  |  |  |
| Q1 | Reference | Reference | Reference | Reference |
| Q2 | 1.85(1.38,2.48) <0.01 | 1.83(1.34,2.50) <0.01 | 1.78(1.28,2.46) <0.01 | 1.68(1.20,2.37) <0.01 |
| Q3 | 1.76(1.31,2.38) <0.01 | 1.69(1.23,2.32) <0.01 | 1.48(1.07,2.05) 0.02 | 1.40(1.00,1.94) 0.05 |
| Q4 | 2.53(1.87,3.43) <0.01 | 2.17(1.60,2.93) <0.01 | 1.91(1.40,2.62) <0.01 | 1.74(1.23,2.45) <0.01 |
| P for Trend | <0.01 | <0.01 | <0.01 | 0.01 |

**Abbreviations**: DM, diabetes mellitus; ACAG, albumin-corrected anion gap; PIR, poverty income ratio; BMI, body mass index; PA, physical activity; CRP, C-reactive protein; CKD, chronic kidney disease; OR, odds ratio; 95% CI, 95% confidence interval.

ACAG quartile cut-off values: Q1≤13.45, 13.45<Q2≤14.65, 14.65<Q3≤15.90, Q4＞15.90.

**Crude model**: Unadjusted.

**Model 1**: Adjusted for demographic factors: age, gender, race, PIR, and education level.

**Model 2**: Adjusted for all variables in Model 1 plus anthropometric and lifestyle factors: BMI, energy intake (log-transformed, kcal/day), potassium intake (g/day), protein intake (g/day), alcohol use, smoking status, and PA (log-transformed).

**Model 3**: Adjusted for all variables in Model 2 plus clinical biomarkers and comorbidities: CRP, hypertension, hyperlipidemia, and CKD.
