# Supplementary material for: Association Between Albumin‐Corrected Anion Gap and Prevalent Prediabetes or Diabetes Mellitus: A Cross‐Sectional Analysis of NHANES 2005–2010
Source: J Diabetes Res. 2026 Sep 18;2026:3632830. doi: 10.1155/jdr/3632830 (PMC13589189; doi:10.1155/jdr/3632830)
Supplement: Supplementary file 2 [file JDR-2026-3632830-s005.docx]

**Table S2.** Comparison of Baseline Characteristics Between Included and Excluded Participants (Restricted to Those with Available Sampling Weights and Complete Data), NHANES 2005–2010.

| Characteristics | Total n=7224 | Exclude n=3287 | Include n=3937 | P-value |
| --- | --- | --- | --- | --- |
| Age, year | 42.32(0.43) | 37.37(0.73) | 45.02(0.48) | <0.01 |
| Gender, n(%) |  |  |  | <0.01 |
| Male | 3676(48.55) | 1554(43.39) | 2122(51.35) |  |
| Female | 3548(51.45) | 1733(56.61) | 1815(48.65) |  |
| Race, n(%) |  |  |  | <0.01 |
| white | 3335(70.02) | 1233(62.78) | 2102(73.96) |  |
| black | 1475(11.34) | 780(14.06) | 695(9.86) |  |
| mexican | 1501( 8.39) | 843(11.33) | 658(6.79) |  |
| other | 913( 10.25) | 431(11.84) | 482(9.39) |  |
| BMI, kg/m2 | 27.98(0.12) | 27.42(0.20) | 28.28(0.13) | <0.01 |
| Education |  |  |  | <0.01 |
| Less than high school | 2854(26.74) | 1992(49.68) | 862(14.26) |  |
| High school or equivalent | 1490(22.00) | 550(20.02) | 940(23.08) |  |
| Greater than high school | 2880(51.26) | 745(30.30) | 2135(62.66) |  |
| PIR^a^, n(%) |  |  |  | <0.01 |
| low | 2311(20.21) | 1297(27.85) | 1014(16.06) |  |
| middle | 2770(36.79) | 1294(40.44) | 1476(34.80) |  |
| high | 2143(43.00) | 696(31.71) | 1447(49.14) |  |
| Prediabetes and DM |  |  |  | 0.52 |
| no | 4816(70.79) | 2317(71.38) | 2499(70.48) |  |
| yes | 2408(29.21) | 970(28.62) | 1438(29.52) |  |

Continuous variables are expressed as unweighted mean (weighted standard deviation); categorical variables are expressed as unweighted count (weighted percentage).

**Abbreviations**: DM, diabetes mellitus; BMI, body mass index; PIR, poverty income ratio.

^a^ PIR was categorized into three groups: low-income (< 1.3), middle-income (1.3–3.5), and high-income (> 3.5).
