# Supplementary material for: Association Between Albumin‐Corrected Anion Gap and Prevalent Prediabetes or Diabetes Mellitus: A Cross‐Sectional Analysis of NHANES 2005–2010
Source: J Diabetes Res. 2026 Sep 18;2026:3632830. doi: 10.1155/jdr/3632830 (PMC13589189; doi:10.1155/jdr/3632830)
Supplement: Supplementary file 3 [file JDR-2026-3632830-s006.docx]

**Table S3**. Reclassification and discrimination indices for PD-DM after adding ACAG to the fully adjusted baseline model.

| Index | Estimate(95%CI) | P-value |
| --- | --- | --- |
| Categorical NRI | 0.08(0.06-0.11) | <0.01 |
| Continuous NRI | 0.42 (0.35–0.48) | <0.01 |
| IDI | 0.04 (0.03–0.06) | <0.01 |

**Abbreviations**: NRI, net reclassification improvement; IDI, integrated discrimination improvement; 95% CI, 95% confidence interval.

**Model 1**: Adjusted for demographic factors: age, gender, race, PIR, and education level.

**Model 2**: Adjusted for all variables in Model 1 plus anthropometric and lifestyle factors: BMI, energy intake (log-transformed, kcal/day), potassium intake (g/day), protein intake (g/day), alcohol use, smoking status, and PA (log-transformed).

**Model 3**: Adjusted for all variables in Model 2 plus clinical biomarkers and comorbidities: CRP, hypertension, hyperlipidemia, and CKD.

**Model definition**: Baseline model contained all covariates in Model 3 without ACAG; Updated model = baseline model + continuous ACAG.

Note: Risk cutoffs of 0, 0.10 and 0.30 were applied for categorical NRI calculation.
