# Supplementary material for: Association Between Albumin‐Corrected Anion Gap and Prevalent Prediabetes or Diabetes Mellitus: A Cross‐Sectional Analysis of NHANES 2005–2010
Source: J Diabetes Res. 2026 Sep 18;2026:3632830. doi: 10.1155/jdr/3632830 (PMC13589189; doi:10.1155/jdr/3632830)
Supplement: Supplementary file 4 [file JDR-2026-3632830-s007.docx]

**Table S4**. Subgroup analysis of the association between ACAG and PD-DM according to menopausal status and age range in females.

| Subgroup | N | OR[95%CI] | P value | P for interaction |
| --- | --- | --- | --- | --- |
| Stratified by menopausal status |  |  |  | 0.5 |
| Non-menopausal | 37 | 1.61(0.81,3.22) | 0.15 |  |
| Menopause | 845 | 1.34(1.23,1.46) | <0.01 |  |
| Stratified by age |  |  |  | 0.9 |
| <45 | 76 | 1.37(0.96,1.97) | 0.08 |  |
| ≥45 | 806 | 1.34(1.22,1.48) | <0.01 |  |

Subgroup analysis based on menopausal status (non-menopausal and menopause) and age range (<45 and ≥45).

**Abbreviations**: OR, odds ratio; 95% CI, 95% confidence interval.
