# Supplementary material for: Association Between Albumin‐Corrected Anion Gap and Prevalent Prediabetes or Diabetes Mellitus: A Cross‐Sectional Analysis of NHANES 2005–2010
Source: J Diabetes Res. 2026 Sep 18;2026:3632830. doi: 10.1155/jdr/3632830 (PMC13589189; doi:10.1155/jdr/3632830)
Supplement: Supplementary file 5 [file JDR-2026-3632830-s008.docx]

**Table S5**. The association between ACAG and DM (n=657) in different models (after excluding individuals with prediabetes [n=781]).

|  | OR(95%CI), P-Value | | | |
| --- | --- | --- | --- | --- |
|  | Crude model | Model 1 | Model 2 | Model 3 |
| ACAG(continuous) | 1.44(1.34,1.55) <0.01 | 1.38(1.28,1.49) <0.01 | 1.30(1.20,1.40) <0.01 | 1.26(1.17,1.36) <0.01 |
| ACAG (quartiles) |  |  |  |  |
| Q1 | Reference | Reference | Reference | Reference |
| Q2 | 1.70(1.23,2.34) <0.01 | 1.60(1.07,2.39) 0.02 | 1.49(0.98,2.26) 0.06 | 1.39(0.91,2.12) 0.12 |
| Q3 | 2.12(1.54,2.91) <0.01 | 1.89(1.31,2.72) <0.01 | 1.55(1.04,2.31) 0.03 | 1.43(0.94,2.18) 0.09 |
| Q4 | 4.81(3.45,6.70) <0.01 | 3.85(2.69,5.50) <0.01 | 2.92(2.05,4.17) <0.01 | 2.55(1.84,3.54) <0.01 |
| P for Trend | <0.01 | <0.01 | <0.01 | <0.01 |

**Abbreviations**: DM, diabetes mellitus; ACAG, albumin-corrected anion gap; PIR, poverty income ratio; BMI, body mass index; PA, physical activity; CRP, C-reactive protein; CKD, chronic kidney disease; OR, odds ratio; 95% CI, 95% confidence interval.

ACAG quartile cut-off values: Q1≤13.45, 13.45<Q2≤14.65, 14.65<Q3≤15.90, Q4＞15.90.

**Crude model**: Unadjusted.

**Model 1**: Adjusted for demographic factors: age, gender, race, PIR, and education level.

**Model 2**: Adjusted for all variables in Model 1 plus anthropometric and lifestyle factors: BMI, energy intake (log-transformed, kcal/day), potassium intake (g/day), protein intake (g/day), alcohol use, smoking status, and PA (log-transformed).

**Model 3**: Adjusted for all variables in Model 2 plus clinical biomarkers and comorbidities: CRP, hypertension, hyperlipidemia, and CKD.
