# Supplementary material for: Association Between Albumin‐Corrected Anion Gap and Prevalent Prediabetes or Diabetes Mellitus: A Cross‐Sectional Analysis of NHANES 2005–2010
Source: J Diabetes Res. 2026 Sep 18;2026:3632830. doi: 10.1155/jdr/3632830 (PMC13589189; doi:10.1155/jdr/3632830)
Supplement: Supplementary file 6 [file JDR-2026-3632830-s010.docx]

Table S1. The definition of covariates.

| Variable Name | Definition/Measurement |
| --- | --- |
| BMI | BMI is calculated by dividing weight by the square of height^[1]^. |
| PIR | Poverty-Income Ratio: Family income relative to poverty threshold (range: 0-5)^[2-3]^. PIR was divided into three strata: low-income (<1.3), middle-income (1.3–3.5), and high-income (>3.5). |
| PA | Physical activity (PA) levels were operationalized using validated metabolic (MET) equivalentalgorithms derived from self-reported exercise duration and intensity metrics^[4-5]^. |
| Drinking | Alcohol consumption status was categorized into five groups: never drinkers (characterized by consuming fewer than 12 alcoholic beverages in their lifetime), former drinkers (referring to individuals who either consumed ≥12 drinks annually in any previous year but abstained in the preceding year, or those who abstained recently despite lifetime consumption exceeding 12 drinks), light drinkers (maintaining daily averages of ≤1 standard drink for women or ≤2 for men over the previous year), moderate drinkers (typically consuming 1-3 daily drinks for women or 2-4 for men averaged over the preceding year), and heavy drinkers (regularly consuming ≥4 daily drinks for women or ≥5 for men throughout the most recent 12-month period)^[6-7]^. |
| Smoking | Behavioral risk factors were assessed through standardized survey instruments administered in mobile examination centers, with tobacco use history categorized as either sustained nicotine consumption (individuals reporting consumption of ≥100 lifetime cigarettes with current use) or abstinence status (those with <100 lifetime cigarettes) ^[8-9]^. |
|  | Clinical biochemistry profiles obtained through standardized venipuncture protocols included inflammatory markers (C-reactive protein, CRP), glucose regulatory hormones (fasting insulin), albumin and serum electrolytes (Na^+^, K^+^, Cl^-^, HCO3^-^). |
| CKD | To diagnose CKD, one of two conditions must exist albuminuria or an eGFR of less than 60 mL/min/1.73 m2. In 2009, the equation for standardized creatinine developed by the Chronic Kidney Disease Epidemiology Collaboration (CKD-EPI) was used to determine eGFR. ACR ≥ 30 mg/g was utilized to characterize albuminuria^[10-11]^. |
| hyperlipidemia | Adult Treatment Panel III (ATP 3) of the National Cholesterol Education Program (NCEP) classified hyperlipidemia as total cholesterol 200 mg/dL, triglycerides 150 mg/dL, HDL 40 mg/dL in males and 50 mg/dL in females, or low-density lipoprotein 130 mg/dL^[12]^. Alternately, persons who reported using cholesterol-lowering drugs were also classified as having hyperlipidemia. |
| hypertension | Hypertension defined as meeting any of the following criteria: (a) self-report of a doctor’s diagnosis of hypertension; (b) current prescription for hypertension medication; (c) three consecutive measurements of systolic blood pressure (SBP) > 140 mmHg and/or diastolic blood pressure (DBP) > 90 mmHg on different days^[13]^. |

Abbreviations: BMI, body mass index; PIR, poverty income ratio; PA, physical activity; CKD, chronic kidney disease.

**References**

[1]	World Health Organization. Obesity: preventing and managing the global epidemic. Report of a WHO consultation. World Health Organ Tech Rep Ser. 2000;894: i-xii, 1-253.

[2]	U.S. Department of Health and Human Services. Annual update of the HHS poverty guidelines--HHS. Notice. Fed Regist. 1998;63(36): 9235-8.

[3]	National Center for Health Statistics (NCHS). NHANES Family Income Questionnaire (2005-2010). Hyattsville, MD: Centers for Disease Control and Prevention. Available at: https://wwwn.cdc.gov/nchs/nhanes/search/variablelist.aspx?Component=Questionnaire&CycleBeginYear=2005. Accessed [2005-2010].

[4]	Ainsworth BE, Haskell WL, Herrmann SD, et al. 2011 Compendium of Physical Activities: a second update of codes and MET values. Med Sci Sports Exerc. 2011;43(8):1575-1581.

[5]	National Center for Health Statistics (NCHS). NHANES Physical Activity Questionnaire (2005-2010). Hyattsville, MD: Centers for Disease Control and Prevention. Available at: https://wwwn.cdc.gov/nchs/nhanes/search/variablelist.aspx?Component=Questionnaire&CycleBeginYear=2005. Accessed [2005-2010].

[6]	National Center for Health Statistics (NCHS). NHANES Alcohol Use Questionnaire (2005-2010). Hyattsville, MD: Centers for Disease Control and Prevention. Available at: https://wwwn.cdc.gov/nchs/nhanes/search/variablelist.aspx?Component=Questionnaire&CycleBeginYear=2005. Accessed [2005-2010].

[7]	National Institute on Alcohol Abuse and Alcoholism (NIAAA). Helping Patients Who Drink Too Much: A Clinician's Guide. Bethesda, MD: National Institutes of Health; 2005.

[8]	National Center for Health Statistics (NCHS). NHANES Smoking - Cigarette Use Questionnaire (2005-2010). Hyattsville, MD: Centers for Disease Control and Prevention. Available at: https://wwwn.cdc.gov/nchs/nhanes/search/variablelist.aspx?Component=Questionnaire&CycleBeginYear=2005. Accessed [2005-2010].

[9]	Centers for Disease Control and Prevention (CDC). Cigarette Smoking Among Adults—United States, 2006. MMWR Morb Mortal Wkly Rep. 2007;56(44):1157-1161.

[10]	Levey AS, Stevens LA, Schmid CH, et al. A new equation to estimate glomerular filtration rate. Ann Intern Med. 2009;150(9):604-612.

[11]	National Kidney Foundation. K/DOQI clinical practice guidelines for chronic kidney disease. Am J Kidney Dis. 2002;39(2 Suppl 1):S1-S266.

[12]	National Cholesterol Education Program (NCEP) Expert Panel on Detection, Evaluation, and Treatment of High Blood Cholesterol in Adults (Adult Treatment Panel III). Third Report of the National Cholesterol Education Program (NCEP) Expert Panel on Detection, Evaluation, and Treatment of High Blood Cholesterol in Adults (Adult Treatment Panel III) final report. Circulation. 2002;106(25):3143-3421.

[13]	Chobanian AV, Bakris GL, Black HR, et al. The Seventh Report of the Joint National Committee on Prevention, Detection, Evaluation, and Treatment of High Blood Pressure: the JNC 7 report. JAMA. 2003;289(19):2560-2572.
